# Cerebrovascular-Specific Extracellular Matrix Bioink Promotes Blood–Brain Barrier Properties

**DOI:** 10.34133/bmr.0115

**Published:** 2024-12-05

**Authors:** Hohyeon Han, Sooyeon Lee, Ge Gao, Hee-Gyeong Yi, Sun Ha Paek, Jinah Jang

**Affiliations:** ^1^ Division of Interdisciplinary Bioscience and Bioengineering, Pohang University of Science and Technology (POSTECH), Pohang 37666, Republic of Korea.; ^2^ Department of Convergence IT Engineering, POSTECH, Pohang 37666, Republic of Korea.; ^3^ School of Medical Technology, Beijing Institute of Technology, Beijing 100081, China.; ^4^ Department of Convergence Biosystems Engineering, College of Agriculture and Life Sciences, Chonnam National University, Gwangju 61186, Republic of Korea.; ^5^ Department of Neurosurgery, Cancer Research Institute, Hypoxia Ischemia Disease Institute, Seoul National University, Seoul 03080, Republic of Korea.; ^6^ Advanced Institutes of Convergence Technology, Seoul National University, Suwon-si, Republic of Korea.; ^7^ Department of Mechanical Engineering, Pohang University of Science and Technology, Pohang 37673, Republic of Korea.; ^8^ Institute of Convergence Science, Yonsei University, Seoul 03722, Republic of Korea.

## Abstract

Chronic neuroinflammation is a principal cause of neurodegenerative diseases such as Alzheimer’s disease and Parkinson’s disease. The blood–brain barrier predominantly comprises endothelial cells, and their intercellular communication with pericytes and other cell types regulates neuroinflammation. Here, we develop a tubular, perfusable model of human cerebrovascular tissues to study neurodegenerative diseases using cerebrovascular-specific extracellular matrix bioink, derived from a complementary blend of brain- and blood-vessel-derived extracellular matrices. The endothelial cells and pericytes in the bioprinted constructs spontaneously self-assemble into a dual-layered structure, closely mimicking the anatomy of the blood–brain barrier. Moreover, the mature cerebrovascular tissue shows physiological barrier functions and neuroinflammatory responses, indicating its potential for developing models of neuroinflammation-related pathologies. Collectively, our study demonstrates that furnishing a cerebrovascular-specific microenvironment can guide the cells to have native-like anatomical relevance and functional recapitulation in vitro.

## Introduction

Chronic neuroinflammation is one of the leading causes of various neurodegenerative diseases such as Alzheimer’s disease, Parkinson’s disease, and amyotrophic lateral sclerosis. It can be triggered by genetic factors, environmental factors, infection, toxic metabolites, or autoimmunity [1]. Understanding how neuroinflammatory responses are regulated is of paramount importance because acute inflammation exerts neuroprotective effects by removing pathogens and toxins, whereas chronic inflammation causes diseases.

The blood–brain barrier (BBB) is a central regulator of neuroinflammation. It is a distinct and continuously evolving microcerebrovascular system composed mainly of tight junctions formed between microvascular endothelial cells in the brain. These endothelial cells interact closely with pericytes, astrocytes, and several other types of cells to maintain BBB integrity [2]. Importantly, endothelial cells and pericytes act as mediators that regulate the propagation of systemic inflammation to the brain at the interface between the brain and peripheral blood flow [3]. In addition, endothelial cells secrete chemokines to facilitate extravasation and the recruitment of peripheral immune cells to injured areas [4,5]. Pericytes also express chemokines and cell-adhesion molecules, present antigens, and display phagocytic abilities [6,7]. Therefore, studying the roles of endothelial cells and pericytes as mediators and participants is crucial for comprehensively understanding the pathophysiology of neuroinflammation.

Tremendous progress has been made in developing in vitro tissue models for mechanistic neuroinflammation studies. Several findings have suggested that integrating multiple cell types into in vitro BBB models can better emulate in vivo conditions. For instance, the triculture BBB model, which combines endothelial cells, astrocytes, and pericytes, is frequently employed to study barrier properties and transport mechanisms. Another model encapsulates endothelial cells, astrocytes, pericytes, and microglia in a 3-dimensional (3D) collagen-based matrix and provides insights into the inflammatory response of the BBB and its implications for resident brain immune cells [8]. Although current BBB models are promising, they lack in vivo-like tubular structures. In terms of cerebrovascular (CBV) function, 3D perfusable and cylindrical channels are important for mimicking fluid flow inside the BBB as well as BBB permeability.

**Fig. 1.**
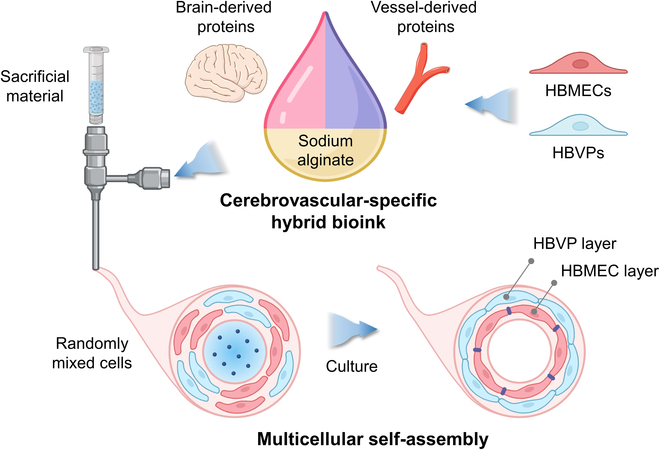
Multicellular self-assembly in 3-dimensional (3D) bioprinted cerebral blood vessel constructs.

Therefore, we developed a model of human tubular CBV tissue that can recapitulate the defensive barrier function and in vivo-like neuroinflammatory responses using 3D bioprinting technology, based on the multicellular self-assembly of human brain microvascular endothelial cells (HBMECs) and human brain vascular pericytes (HBVPs) (Fig. 1). To provide microenvironmental cues that facilitate endothelial maturation and multicellular interactions during neuroinflammation in a cerebro-blood vessel model, we developed a novel biomaterial that closely recapitulated the CBV microenvironment. For the first time, we introduced a complementary mixture of a vessel- and a brain-derived decellularized extracellular matrix (VdECM and BdECM, respectively) as a CBV-specific decellularized extracellular matrix (CBVdECM). Using this original biomaterial, we established a single-step method for fabricating CBV tissue constructs and a supporting chamber via coaxial printing with a multiprinter head system.

## Materials and Methods

### BdECM hydrogel preparation

First, we decellularized porcine brain tissue, following a previously described protocol, with slight modifications [9]. We prepared porcine cephalic parts cut along the sagittal plane from an online butcher shop (PIGNARA, www.pignara.com), then isolated the cortices from the subarachnoid space, and stored them at −80 °C until use. For decellularization, we cut the tissue into 5 mm × 5 mm × 5 mm pieces and washed them sequentially in volumes of decellularization solutions that were 10 times larger than that of the tissue. All the solutions contained 1% penicillin/streptomycin (HyClone, South Logan, Utah, USA). The washing steps were as follows: (a) deionized water (DIW) for 48 h, (b) 0.5% sodium dodecyl sulfate (BioShop, Burlington, Canada) in DIW for 24 h, (c) phosphate-buffered saline (PBS; Tech & Innovation [T&I], Chuncheon, Korea) for 15 min, (d) 50 U/ml DNase (Sigma-Aldrich, St. Louis, Missouri, USA) in 1 M NaCl solution for 12 h at 37 °C, (e) 0.5% Triton X-100 (Biosesang, Yongin, Korea) in DIW for 48 to 96 h, (f) PBS for 15 min, and (g) 0.1% peracetic acid (Taesung International, Seoul, Korea) in 4% ethanol (Samchun, Seoul, Korea) for 2 h. Finally, the tissues were washed at least 8 times with PBS and twice with DIW without penicillin/streptomycin. All solutions were rocked at 15 rotations/min (RPM) and 4 °C (except for step d). After centrifugation at 1,500 RPM for 5 min, each wash solution was replaced by draining the supernatant. We lyophilized the decellularized brain tissue and stored it at −80 °C before solubilization. We prepared the BdECM hydrogel by solubilizing the required amount of BdECM powder in 0.1 N HCl (Sigma-Aldrich) solution containing pepsin (Sigma-Aldrich) at a concentration equivalent to one-tenth of the decellularized extracellular matrix (dECM) weight. The mixture was stirred at 1,000 RPM for 72 h. We used 1 N NaOH (Biosesang) to terminate pepsin digestion and neutralize the acidic solution. Finally, we added 10× PBS to the BdECM pregel solution to maintain osmotic balance.

### VdECM hydrogel preparation

First, porcine aorta tissues were decellularized following a previously described protocol [10], with slight modifications. Fresh porcine descending aortas were obtained from a local slaughterhouse and stored at −80 °C until use. The tissue was minced into 2 mm × 2 mm × 2 mm cubes before decellularization. Subsequently, we washed the tissue in decellularization solutions that were 80 times larger than the tissue volume. The wash steps were as follows: (a) DIW for 6 h, (b) 0.3% sodium dodecyl sulfate (BioShop) in PBS (T&I)/ethylenediaminetetraacetic acid (EDTA; T&I) for 24 h, (c) PBS for 24 h, (d) 0.5% Triton X-100 (Biosesang) in PBS/EDTA for 24 h, (e) PBS for 24 h, (f) 75 U/ml DNase (Sigma-Aldrich) in 50 mM MgCl_2_ in PBS for 24 h at 37 °C, (g) PBS for 24 h, and (h) 0.1% peracetic acid (Taesung International) in 4% ethanol (Samchun) in PBS for 4 h. The tissues were then washed thrice with PBS and twice with DIW. All solutions were stirred at 120 RPM at room temperature (20 °C) (except for step f). The decellularized blood vessel tissues were lyophilized and stored at −80 °C before solubilization. We prepared a VdECM hydrogel by digesting the required amount of VdECM particles in 0.5 M acetic acid solution (Merck Millipore, Darmstadt, Germany) containing pepsin (Sigma-Aldrich) at a concentration equivalent to one-tenth of the dECM weight. The mixture was stirred for 168 h at 800 RPM. To terminate the pepsin digestion, the acidic solution was neutralized with 10 N NaOH (Biosesang). Finally, we added 10× PBS to the VdECM pregel solution to support osmotic balance. For our comparative study, we prepared a collagen pregel solution in the same way, digesting dry collagen (Dalim Tissen Co., Ltd., Seoul, Korea) in 0.5 M acetic acid and then adjusting the solution to have physiological conditions in terms of pH and osmotic balance.

### Biochemical assay

We quantified the decellularization efficiency by measuring the amounts of DNA and glycosaminoglycans (GAGs) remaining in the decellularized tissues and compared them with those in the native tissues. The double-stranded DNA contents were quantified using the Quant-iT PicoGreen dsDNA Reagent and Kits (Invitrogen, Carlsbad, California, USA), following the manufacturer’s instructions. GAG contents were evaluated by quantifying the number of sulfated GAGs using 1,9-dimethyl methylene blue solution [11]. First, all dECMs and native tissues were digested in 1 ml of papain solution (125 μg ml^−1^ papain in 0.1 M sodium phosphate with 5 mM Na_2_·EDTA and 5 mM cysteine·HCl at pH 6.5) for 16 h at 60 °C. Next, a standard curve was constructed using chondroitin sulfate A, and the absorbance at 525 nm was measured using a microplate reader after reaction with the 1,9-dimethyl methylene blue solution.

### Cell culture

Primary HBMECs (Innoprot, Bizkaia, Spain) were propagated in T75 tissue culture plates coated with fibronectin (Corning Inc., Corning, New York, USA) and maintained in endothelial cell medium (Innoprot). Primary HBVPs (ScienCell, Carlsbad, California, USA) were maintained in pericyte medium (ScienCell). Primary cells were used in our experiments from passages 4 to 7.

### Proteomic analysis

Proteomic analysis was conducted using liquid chromatography with tandem mass spectrometry. BdECM and VdECM samples were digested into peptides via in-solution digestion. For each sample, 8 M urea in 100 mM ammonium bicarbonate was added to a final concentration of at least 6 M, and the mixture was incubated for 20 min at room temperature. The proteins were reduced via alkylation with 10 mM dithiothreitol and denatured with 30 mM iodoacetamide. Then, the samples were trypsinized overnight at 37 °C. The trypsin reaction was quenched with 0.4% trifluoroacetate, and the peptides were desalted using a C18 Harvard macro spin column. The resulting peptides were dried, resuspended in water containing 0.1% formic acid, and analyzed using a Q Exactive Orbitrap hybrid mass spectrometer coupled with a nanoACQUITY ultraperformance liquid chromatography instrument (Waters, Manchester, UK). The peptides were eluted from a trap column and ionized using a nanospray ionization system coupled with an in-house column (100 cm × 75 μm) packed with 2-μm C18 particles at an electric potential of 2.0 kV. The maximum ion injection time for tandem mass spectrometry was set to 60 ms, with a resolution of 17,500. The dynamic exclusion time was set to 30 s. Raw files were searched against the UniProt Database using the MaxQuant software (version 1.5.1.2). The proteomic compositions were identified by searching a sequence database with Proteome Discovery (version 2.2).

### Rheological assessments

Rheological properties were measured using an Advanced Rheometric Expansion System (TA Instruments, New Castle, Delaware, USA) with a 20-mm-diameter plate geometry. The viscosities of the 1.5% BdECM, VdECM, and CBVdECM hydrogels were measured via steady shear-sweep analysis at 4 °C. The dynamic moduli were measured by dynamic frequency-sweep analysis after incubation for 1 h at 37 °C.

### Tube formation assay

We coated μ-slide angiogenesis plates (ibidi, Martinsried, Germany) with cold Matrigel (Corning Inc.). After incubating them for 1 h at 37 °C, HBMECs and HBVPs (2 × 10^5^ cells/ml) were seeded into Matrigel-coated wells. After 15 h, we captured images of each entire well at low magnification (×4). The total tube length and number of branching points formed by cells observed in each image field were quantified using the WimTube software (Onimagin Technologies SCA, Córdoba, Spain).

### Cell viability and proliferation

We evaluated cell viabilities using 2 × 10^6^ ml^−1^ HBMECs and HBVPs encapsulated in dECM hydrogels. The cell suspensions were mixed with 1.5 wt. % dECM hydrogels and gelated by incubation at 37 °C for 30 min. We added a cell medium (1:1 ratio of endothelial cell medium to pericyte medium) and cultured them for 14 d. Cell viability was assessed on days 1, 7, and 14 using a LIVE/DEAD Cell Viability Assay Kit (Thermo Fisher Scientific, Waltham, Massachusetts, USA), following the manufacturer’s instructions. Cell proliferation was evaluated by measuring the metabolic activities of viable cells using Cell Counting Kit-8 (CCK-8; Dojindo, Tokyo, Japan). We diluted the CCK-8 reagent in a fresh culture medium at a 1:10 ratio and replaced the medium with an agent-loaded medium. This agent generated formazan via cellular metabolism in the presence of viable cells. After incubation for 2 h at 37 °C, the culture medium (containing formazan) was transferred from the culture plate to a new transparent 96-well plate in triplicate. Absorbance was measured at 450 nm. The CCK-8 assays were performed simultaneously on days 1, 4, 7, 10, and 14.

### Neuroinflammation induction

To mimic neuroinflammation in the encapsulated cells and bioprinted CBV tissue constructs, we applied 100 ng/ml tumor necrosis factor-α (TNF-α; 210-TA, R&D Systems, Inc., Minneapolis, Minnesota, USA) or interleukin-1β (IL-1β) (201-LB, R&D Systems, Inc.) for 24 h.

### Differential expression of human inflammatory cytokines

To quantify the levels of inflammatory cytokines in culture supernatants after inducing neuroinflammation in cells embedded in collagen, BdECM, VdECM, and CBVdECM, we performed cytokine arrays using Proteome Profiler Human Cytokine Array Kit (ARY005B, R&D Systems, Inc.). First, we applied a blocking buffer for 1 h to block the membranes before detecting 36 different inflammatory cytokines. Next, we reconstituted a cocktail containing detection antibodies and mixed them with the samples. The sample–antibody mixtures were added to the membranes and incubated overnight on a rocking platform shaker at 4 °C. On the next day, each membrane was placed in an individual plastic container and washed thrice with wash buffer for 10 min on a rocking platform shaker. Streptavidin–horseradish peroxidase was then added to the membranes, and they were further incubated for 30 min at room temperature in a rocking platform shaker. After washing, each membrane was incubated with 1 ml of a chemireagent mix (included in the kit) for 1 min, as described previously. Finally, we exposed the membranes to a ChemiDoc reader, collected the image files, and analyzed their intensities.

### Bioink preparation

The bioink employed for coaxial printing was formulated using a 6 wt. % sodium alginate solution, which was prepared by dissolving alginic acid sodium salt from brown algae (Sigma-Aldrich) in DIW, 3 wt. % CBVdECM, and 10× Dulbecco’s modified Eagle’s medium. The final bioink contained 1× Dulbecco’s modified Eagle’s medium, 2.4% CBVdECM, and 0.6% sodium alginate. Mechanical agitation was performed overnight at room temperature to obtain a homogeneous solution. Before printing, we embedded HBMECs and HBVPs (1 × 10^7^ cells ml^−1^ each) in the neutralized bioink. Pluronic F-127 (PF-127; Sigma-Aldrich) was dissolved in a 100 × 10^−3^ M CaCl_2_ solution at a 35 wt. % concentration to obtain the CPF-127 hydrogel.

### Three-dimensional bioprinting

We used a 3D-printing system (3DX Bioprinter, T&R Biofab, Siheung, Korea) and metal precision nozzles (HN-0.4N, Musashi Engineering, Tokyo, Japan) to print polyethylene vinyl acetate (PEVA; PolyScience, USA) chambers and coaxial nozzles (14G/18G, Ramé-Hart Instrument Co., Succasunna, New Jersey, USA) to print the CBV tissue constructs following a previous method [10,12], with slight modifications. First, we printed side-media chambers using PEVA to perfuse the cell culture medium or fluorescent tracers into the luminal area of the cerebral blood vessel models. PEVA was kept in a cartridge at 110 °C, and the chambers were printed at an environmental temperature of 25 °C. The cerebral blood vessel model was printed in the chamber. Next, the cell-laden CBVdECM bioink and CPF-127 were coextruded from the shell and core of the coaxial nozzle at respective temperatures of 4 and 37 °C and respective pneumatic pressures of 30 and 110 kPa in order to fabricate the tubular structure. After printing, we used 10 wt. % low-melting-point agarose (Invitrogen), preserved at 60 °C after sterilization in an autoclave, to tightly seal the gap between the printed cerebral blood vessel construct and the inlet medium chamber.

### Validation of compartmentalization

We validated core and shell compartmentalization using fluorescent beads with different colors (Spherotech Inc., Lake Forest, Illinois, USA) in the core and shell. After printing, we conducted confocal imaging to evaluate the compartmentalization.

### Immunostaining

To examine the cellular morphology (including junctional protein expression and the multicellular self-assembly of CBV tissue constructs) after culture, we conducted immunofluorescence staining. Antibodies against vascular endothelial cadherin (VE-cadherin; a junctional protein; catalog number ab33168, Abcam, Cambridge, Massachusetts, USA), cluster of differentiation 31 (CD31; a marker of endothelial cells; ab28364, Abcam), platelet-derived growth factor receptor beta (PDGFR-β; a marker of pericytes; ab69506, Abcam), and filamentous actin (*F*-*actin; a marker of microfilaments;* F432, *Invitrogen*) were used. The cultured tissue samples used for the biological evaluation of biomaterials (e.g., collagen, BdECM, VdECM, and CBVdECM) or CBV tissue constructs were fixed with 4% paraformaldehyde in PBS for 15 min and then washed with PBS. Immunostaining was performed after permeabilization with PBS containing 0.1% Triton X-100 (Sigma-Aldrich) for 15 min and blocking for 1 h using PBS containing 5% normal goat serum. Antibodies against VE-cadherin (1:100), PDGFR-β (1:100), CD31 (1:50), and F-actin (1:40) were diluted in PBS containing 1% bovine serum albumin at specific ratios. The diluted antibodies were added to the constructs and incubated overnight at 4 °C. The constructs were stained with secondary antibodies conjugated with Alexa Fluor-488 and Alexa Fluor-594 and analyzed by confocal microscopy.

### Permeability assay

We performed permeability testing to confirm the barrier function of the CBV tissue constructs. After printing the chamber and the cerebral blood vessel model, the remaining space in the middle chamber was filled with 1.5 wt. % collagen to support the CBV tissue construct, which generated a 3D matrix after sufficient gelation during 7 d in culture. Next, 1 × 10^−5^ M fluorescein isothiocyanate (FITC)-conjugated dextran (10 or 40 kDa, Sigma-Aldrich) was added to the medium and used as a fluorescent tracer. Subsequently, 800 μl of dextran solution was injected into the inlet, and the chamber was gently rocked at 5 RPM on a shaker (Daihan Scientific, Wonju, Korea) for continuous perfusion. The diffusion patterns were recorded after 10 and 30 min using the NIS-Elements Advanced Research software with a fluorescence microscope (ECLIPSE Ti, Nikon, Tokyo, Japan). We then analyzed the diffusional permeability by quantifying the mean changes in the fluorescence intensity in the entire region over time using Eq. 1:

$$\mathrm{Pd}=\frac{1}{\left(I1-\mathrm{Ib}\right)}\left(\frac{I2-I1}{t}\right)\frac{d}{4}$$ (1)

where *Pd* is the coefficient of diffusional permeability; *I*1 and *I*2 are the average fluorescence intensities initially and at certain time points (*t*; 10 or 30 min in our study), respectively; *Ib* is the background intensity without FITC–dextran; and *d* is the channel diameter [13].

### Enzyme-linked immunosorbent assay analysis of secreted inflammatory cytokines

To measure intercellular adhesion molecule 1 (ICAM-1) expression and evaluate its regulatory effect upon regulated on activation, normal T cell*–*expressed and secreted (RANTES) activation in the culture supernatant, we used a commercially available enzyme-linked immunosorbent assay kit against human ICAM-1 and human RANTES (catalog numbers DY720 and DY278, respectively, R&D Systems, Inc.).

### Statistical analysis

All data are presented as mean ± standard deviation. Statistical significance was determined using one-way analysis of variance. *P < 0.05, **P < 0.001, and ***P < 0.0001. Prism 7 (GraphPad Software, San Diego, California, USA) was used for the statistical analyses.

## Results

### The complementary blend of 2 tissue-derived biomaterials mimics the CBV biochemical niche

**Fig. 2.**
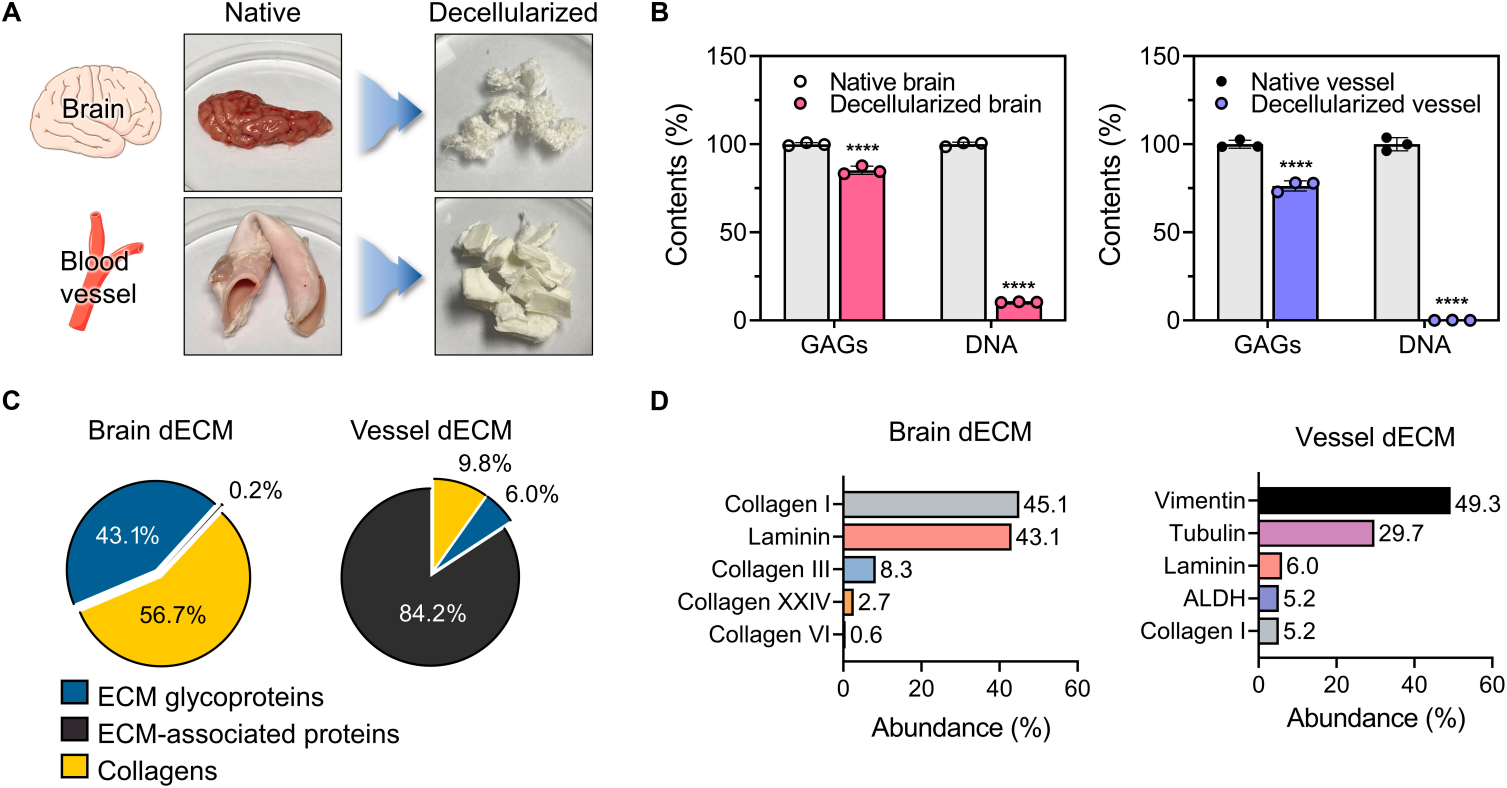
Biochemical validation and proteomic characterization of the brain-derived decellularized extracellular matrix (BdECM) and vessel-derived decellularized extracellular matrix (VdECM). (A) Native and decellularized porcine brain and blood vessel tissues. (B) Glycosaminoglycan (GAG) and DNA contents of native tissues, the BdECM, and the VdECM (*n* = 3 per group; ****P < 0.0001; 2-way analysis of variance [ANOVA]). Data represent mean ± standard deviation (SD). (C) Characterization of the extracellular matrix (ECM) components of each decellularized extracellular matrix (dECM) using proteomic analysis. (D) Top 5 most abundant proteins in the BdECM and VdECM.

We hypothesized that novel CBV-specific biomaterials are necessary to replicate the native BBB microenvironment and optimize cell function. We developed a CBVdECM, comprising a blend of essential extracellular matrices (ECMs) from brain and blood vessels. The major components of the brain- and aorta-specific proteins were obtained by decellularizing porcine brain and aorta tissues (Fig. 2A).

Decellularizing porcine brain and aorta tissues drastically decreased the DNA contents of the BdECM and VdECM to 29.19 ± 0.40 ng/mg (native brain: 280.45 ± 3.43 and 1.68 ± 1.48 ng/mg; native aorta: 1,584.13 ± 59.65 ng/mg) while preserving 84.65% ± 2.51% and 72.99% ± 4.33% of their GAG contents when compared with those in the native tissues, respectively (Fig. 2B and Table S1).

To characterize the ECM components of the BdECM and VdECM, we conducted proteomic analysis. The peptide abundances of core matrisome proteins (collagen, glycoproteins, and proteoglycan) and ECM-associated proteins [14] demonstrated distinct compositional differences between BdECM and VdECM (Fig. 2C). Among the top 5 most abundant proteins preserved in the BdECM or VdECM, the most abundant components in the BdECM were collagen and laminin (Fig. 2D), which are the major constituents of the BBB basement membrane and are essential for tight junction formation and structural integrity [15]. In contrast, the most abundant components preserved in the VdECM were vimentin and tubulin, which regulate endothelial sprouting during vasculogenesis [16–19].

### The cerebrovascular dECM niche induces physiologically relevant cellular responses

**Fig. 3.**
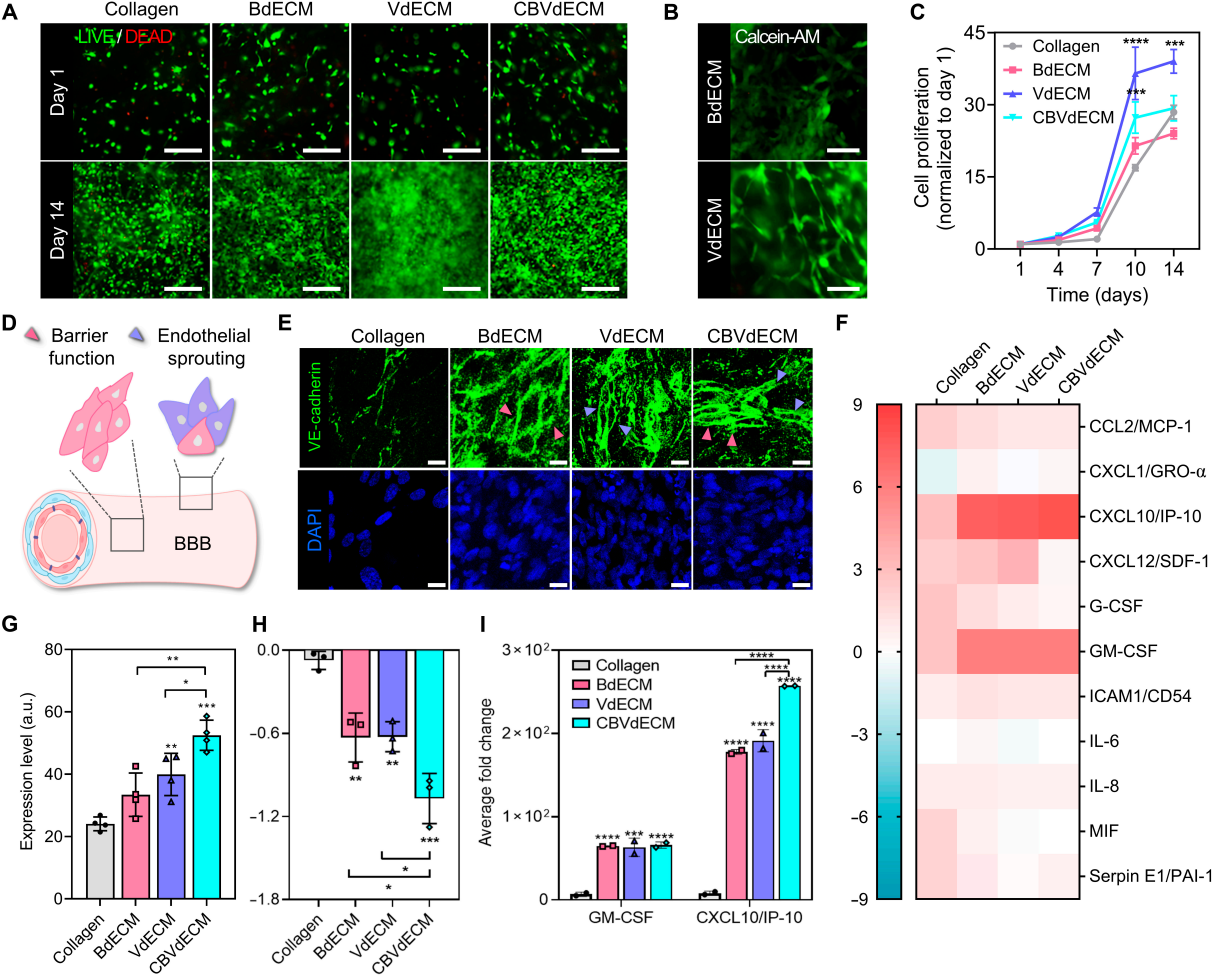
Recapitulation of cerebral-blood-vessel-specific cellular functionality and neuroinflammatory responses using 4 types of ECM biomaterial inks. (A) Validation of the cellular compatibility of different biomaterial inks. LIVE/DEAD (green/red) assay images of cells encapsulated in collagen, the BdECM, the VdECM, and the cerebrovascular-specific decellularized extracellular matrix (CBVdECM) after 14 d in culture (scale bars: 200 μm). (B) Overall morphological differences in human brain microvascular endothelial cells (HBMECs; 1 × 10^6^ cells/ml) encapsulated in 1% BdECM or VdECM owing to proteomic differences (green: calcein-AM; scale bars: 100 μm). (C) Proliferation of cells encapsulated in ECM biomaterial inks. Data represent mean ± standard error of the mean (SEM, *n* = 4 per group; ***P < 0.001; ****P < 0.0001; 2-way ANOVA). (D) Schematic illustration of cerebral-blood-vessel-specific cellular functionality in the cerebrovascular (CBV) constructs. (E) Basement membrane-like structures (pink arrows) and endothelial sprouting morphologies (purple arrows) of endothelial junctions (vascular endothelial cadherin [VE-cadherin], green) formed in 1.5% collagen, BdECM, VdECM, or CBVdECM after 14 d in culture (HBMECs + human brain vascular pericytes [HBVPs], 2 × 10^6^ cells/ml; scale bars: 100 μm). (F) Heatmap results of an inflammatory cytokine array after inducing neuroinflammation by tumor necrosis factor-α treatment. Each biomaterial showed different trends in their fold changes in inflammatory cytokines. (G) Quantification of the results presented in (E). Data represent mean ± SEM (*n* = 4 per group; *P < 0.05; **P < 0.01; ***P < 0.001; 2-way ANOVA). (H) Significant junctional down-regulation following intensive up-regulation of cytokines in the CBVdECM. Data represent mean ± SEM (*n* = 3 per group; *P < 0.05; **P < 0.01; ***P < 0.001; 2-way ANOVA). (I) Fold changes in 2 representative neuroinflammatory cytokines, namely, granulocyte-macrophage colony-stimulating factor (GM-CSF) and C-X-C motif chemokine ligand 10 (CXCL10)/interferon-gamma-induced protein 10 (IP-10). Data represent mean ± SEM (*n* = 2 per group; ***P < 0.001; ****P < 0.0001; 2-way ANOVA).

During the early stages of vascular network development, tubelike network formation is guided by intrinsic cellular self-assembly [20,21]. Therefore, to determine the optimal co-culture ratio that could promote de novo vascularization, we performed tube formation assays in Matrigel-coated plates. Various HBMEC:HBVP co-culture ratios were tested, and a 1:1 ratio was found to be optimal (Fig. S1). We then encapsulated HBMECs and HBVPs at a 1:1 ratio in collagen, the BdECM, the VdECM, and the CBVdECM to evaluate their biocompatibility, barrier function, and vascularization in each biomaterial. Although all groups showed similar cell viabilities of >95% after 14 d in culture (Fig. 3A), the VdECM and CBVdECM groups showed notably higher proliferation rates than the collagen and BdECM groups (Fig. 3C). Calcein-AM staining demonstrated a drastic difference in the morphology of HBMECs encapsulated in the BdECM or VdECM (1% by weight). The cells in the BdECM exhibited tight cell–cell contacts when they formed tube morphologies related to de novo vascularization in the VdECM (Fig. 3B).

Next, we investigated how the developed combination of biomaterials affected CBV function with a 3D dome model, especially tight junction formation. A 1:1 mixture of HBMECs and HBVPs was embedded in collagen, the BdECM, the VdECM, or the CBVdECM and cultured for 14 d. We confirmed that the CBVdECM simultaneously enhanced cell–cell contact barrier function (Fig. 3D, pink arrow) and endothelial sprouting (Fig. 3D, purple arrow). Specifically, the expression of the tight junction protein, VE-cadherin, demonstrated notably different morphologies with each biomaterial. In the BdECM and CBVdECM groups, a linear pattern of junctional fusion was observed owing to the maturation of cell–cell contacts (Fig. 3E, pink arrows). Moreover, we observed endothelial-tip cell formation and directed ECM invasion in the VdECM and CBVdECM groups (Fig. 3E, purple arrows), which is the initial step of de novo vascularization [22]. Furthermore, the CBVdECM group showed the highest expression of VE-cadherin, which is essential for tight barrier formation in cerebral blood vessels (Fig. 3G). These morphological differences between the BdECM and VdECM groups may have resulted from their distinct proteomic compositions (Fig. 2D). Collagen and laminin, which are abundant in the BdECM, can support cell–cell contact maturation, whereas vimentin and tubulin (abundant in the VdECM) encourage endothelial sprouting and primitive vascular plexus formation during vascular network development [16–19].

Subsequently, we evaluated differences in the neuroinflammatory responses of cells encapsulated in each biomaterial (collagen, BdECM, VdECM, and CBVdECM). Junctional breakdown and inflammatory cytokine expression levels were measured after inducing inflammation by treatment with TNF-α. Each biomaterial showed different trends in terms of the fold changes in inflammatory cytokine expression levels observed. The CBVdECM group showed markedly higher junctional breakdown than the other groups (Fig. 3H). Mimicking junctional breakdown is important for modeling the facilitated transport of immune cells into brain sites for neuroprotection under inflammatory conditions [2]. Among the 11 up-regulated cytokines observed, 8 were related to neuroinflammation; these included 4 cytokines related to immune activation and differentiation (granulocyte-macrophage colony-stimulating factor [GM-CSF], granulocyte colony-stimulating factor, interleukin 6, and C-X-C motif chemokine ligand 1/growth regulated oncogene alpha) and 4 related to immune cell migration and microglial cell clustering (C-X-C motif chemokine ligand 1 [CXCL10]/interferon-gamma-induced protein 10 [IP-10], C-C motif ligand 2/monocyte chemoattractant protein-1, ICAM-1/cluster of differentiation 54, and interleukin 6) (Fig. 3F). Notably, GM-CSF and CXCL10/IP-10 were notably up-regulated in the dECM group (Fig. 3I). GM-CSF exerts neuroprotective effects by inducing myeloid cell activity in injured neurons. In particular, GM-CSF induces tight junction disassembly in epithelial layers by down-regulating zonula occludens-1 and claudin-5 expression via transcriptional modulation and ubiquitination [23]. The significant up-regulation of GM-CSF under induced inflammation indicates that dECMs have sufficient potential to recapitulate the junctional breakdown phenomenon in vitro, similar to in vivo neuroinflammation. In addition, CXCL10/IP-10 initiates a cascade that down-regulates junctional protein expression, increases BBB permeability, and stimulates inflammatory cell infiltrations [24,25]. Interestingly, CXCL10/IP-10 expression was also considerably higher in the dECM group than in the collagen group, with the CBVdECM showing the most significant fold change (Fig. 3I).

Taken together, these data showed that the CBVdECM induced superior functionality in cells, in terms of proliferation rates, morphology, junctional protein expression levels, and pathophysiological responses, when compared with other ECM biomaterials. In particular, the CBVdECM group showed outstanding performance when compared with type I collagen, which has been frequently used to model the BBB [26–28], largely owing to its compositional diversity. Compared with single-ECM materials (such as collagen), CBVdECM encompasses additional crucial components of the brain and vascular tissues, which help promote various CBV developments. Accordingly, we fabricated cerebral blood vessel constructs using the CBVdECM as the bioink material.

### CBV models are fabricated using 3D bioprinting with versatile printing parameter control

**Fig. 4.**
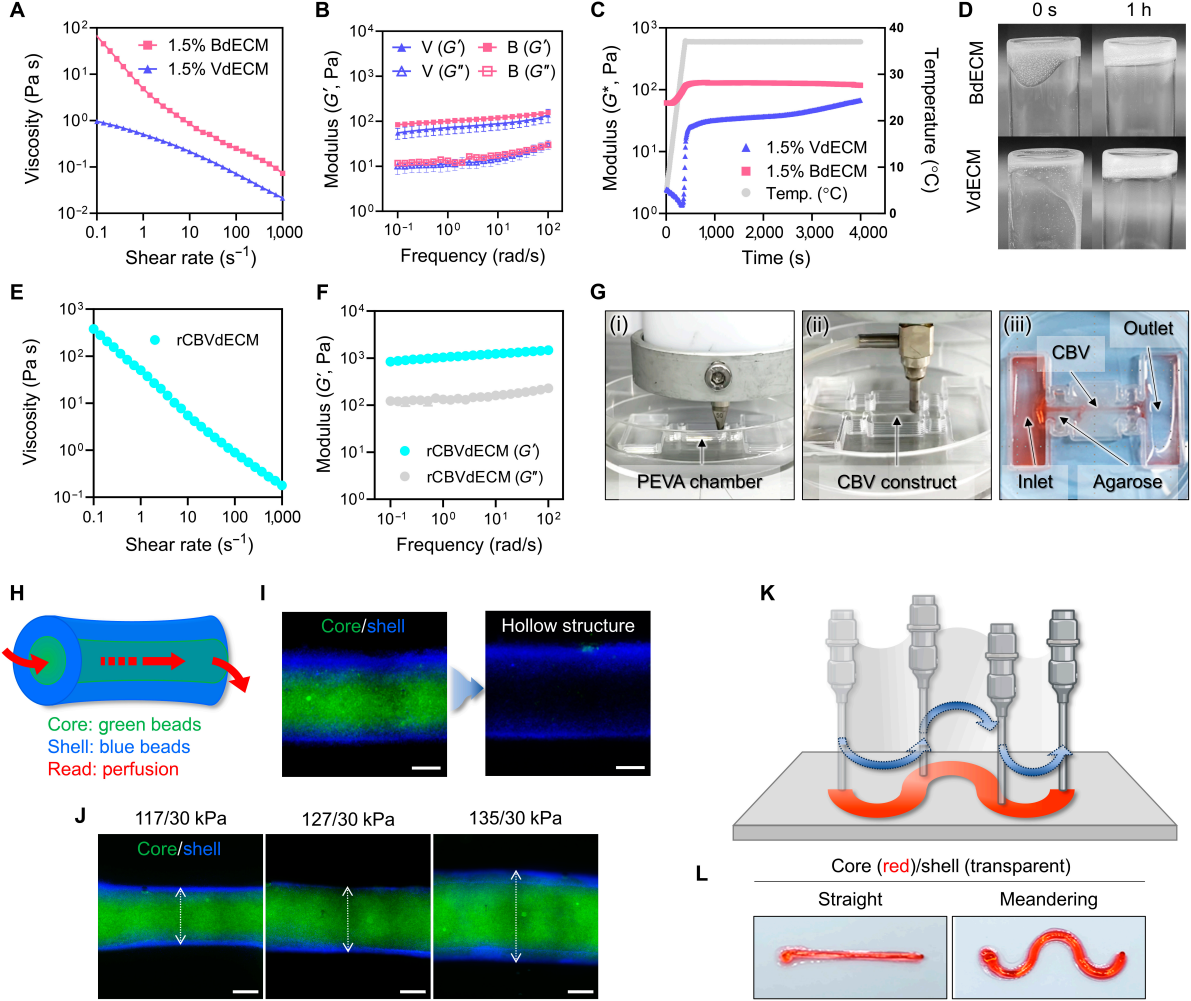
Rheological validation of CBVdECM bioink and 3D bioprinting of CBV constructs. (A) Viscosity of 1.5% BdECM and VdECM hydrogels exhibiting shear-thinning behavior. (B) The complex moduli of the cross-linked BdECM (abbreviated as “B”) and VdECM hydrogels (abbreviated as “V”) showed higher storage moduli than loss moduli. (C) Gelation kinetics of the BdECM and VdECM hydrogels from 4 to 37 °C. (D) Sol–gel behavior of the BdECM and VdECM hydrogels before and after cross-linking for 1 h at 37 °C. (E and F) Viscosity (E) and complex modulus (F) values of cross-linked, reinforced CBVdECM bioink (abbreviated as “rCBVdECM”). (G) Automatic one-step printing process of polyethylene vinyl acetate (PEVA) chambers and CBV constructs: (i) printing of the PEVA chamber, (ii) coaxial printing of CBV constructs, and (iii) luminal perfusion of CBV constructs. (H) Schematic illustration of the luminal perfusion process used to remove the core sacrificial material and obtain a hollow structure. (I) Visual validation of perfusable CBV constructs using fluorescent beads with different colors. (J) Controlling the construct diameters by changing the core/shell pneumatic pressure (scale bars: 500 μm). (K and L) Schematic representation of the structural printing versatility (K) and actual results (L) (straight and meandering lines). Data represent mean ± SEM. All experiments were performed in triplicate.

Next, the mechanical properties of the BdECM and VdECM hydrogels were analyzed to identify a biofabrication window within which the dECM hydrogels reliably showed appropriate 3D printability and cell viability. First, we measured the viscosities of the dECM hydrogels in the pregel state to examine whether they possessed the requisite printability as bioinks (Fig. 4A). Bioinks must exhibit proper rheological properties such as shear-thinning behavior, which can relieve shear stress and help maintain the viability of encapsulated cells when passing through the printing nozzle.

The viscosities of both 1.5 wt. % BdECM and VdECM continuously decreased as the shear rate increased, thereby mimicking shear-thinning behavior. The viscoelastic behaviors of the BdECM and VdECM exhibited greater storage moduli than loss moduli in the given oscillation range (Fig. 4B). The fibrillar components in the dECM hydrogels assembled sufficiently strongly after gelation at physiological temperatures, which provided a stable environment for cells to adhere and grow. Finally, we characterized the gelation kinetics of dECM hydrogels (Fig. 4C). Both the BdECM and VdECM hydrogels exhibited stable thermal cross-linking after incubation at 37 °C for 1 h. We confirmed that the dECM biomaterials exhibited sol–gel transition behavior at physiological temperatures (Fig. 4D).

Based on our characterization of the BdECM and VdECM hydrogels as printable biomaterials, we assessed the printability of the CBVdECM for its applicability with a previously developed coaxial printing strategy. We improved the cross-linking efficiency of the CBVdECM by reinforcing it with alginate (2.4 wt. % CBVdECM + 0.6 wt. % alginate) to induce instantaneous cross-linking of a printed tubular construct to maintain the vessel patency. The pregel state of the reinforced CBVdECM (rCBVdECM) bioink exhibited shear-thinning behavior (Fig. 4E). The storage modulus of the rCBVdECM bioink was greater than its loss modulus, which helped retain its 3D structure after printing (Fig. 4F).

Finally, we optimized the printing process to successfully fabricate tubular cerebral blood vessel constructs using a one-step process (Fig. 4G and Movie S1). First, we designed a chamber housing that structurally supported the cerebral blood vessel construct and enabled continuous pumpless medium flow during the culture period, as shown in Fig. 4G (i). The chamber was printed using the biocompatible polymer PEVA. Figure 4G (ii) shows a fabricated cerebral blood vessel construct after coaxial printing in the middle of a PEVA chamber. PF-127 was selected as the core material because it is soluble and easily dissolves in the culture medium. In addition, by adding CaCl_2_ to PF-127, we obtained a useful sacrificial material (CPF-127), enabling ionic gelation of alginate in the rCBVdECM bioink and thereby structurally supporting simultaneous core/shell compartmentalization during printing. Embedding both ends of the CBV tissue construct with a natural polymer, agarose, resulted in a perfusive vessel structure, as shown in Fig. 4G (iii). Core CPF-127 was easily removed during incubation of the printed tissues (Fig. 4H and I). The combination of rCBVdECM and CPF-127 enhanced the robustness of the cerebral blood vessel model. For example, the structural and diameter versatility of the cerebral blood vessel constructs was enhanced by simply controlling the printing path or pneumatic pressure changes while maintaining compartmentalized core/shell structures (Fig. 4J to L).

### Spontaneous self-assembly of bioprinted CBV models induces dual-layered lumen formation

**Fig. 5.**
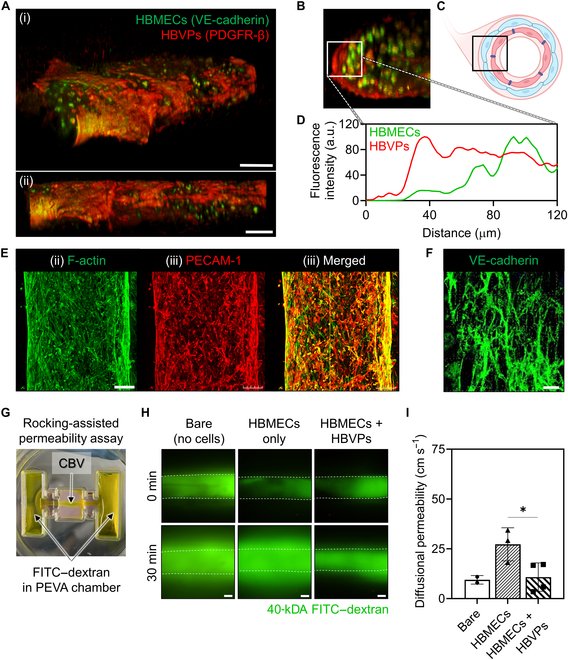
Multicellular self-assembly of 3D bioprinted CBV constructs. (A and B) Postculture 3D reconstructed confocal microscopy images showing multicellular self-assembly of the printed CBV constructs: HBMECs (VE-cadherin, green) and HBVPs (platelet-derived growth factor receptor beta [PDGFR-β], red); scale bars: 200 μm. (C) Schematic representation of HBMEC and HBVP compartmentalization. (D) Fluorescence intensity plot of the cells in the white box in (B), indicating the compartmentalization of HBVPs (red) and HBMECs (green). (E) Filamentous actin (F-actin) and platelet endothelial cell-adhesion molecule-1 (PECAM-1) expression in mature CBV constructs (scale bars: 200 μm). (F) Morphology of junctional proteins (scale bar: 40 μm) in mature CBV constructs. (G) Permeability assay of the CBV constructs cast in PEVA chambers using fluorescein isothiocyanate (FITC)-labeled dextran. (H) Permeability differences between the bare, HBMEC-only, and HBMEC + HBVP self-assembled constructs (green: 40-kDa FITC–dextran; scale bars: 200 μm). (I) Calculated diffusional permeability values for the constructs shown in (H). Data represent mean ± SEM (*n* = 2, 3, and 4 respectively; *P < 0.05; unpaired Student *t* test).

Employing the ability of cells to self-assemble into functional tissues is one of the ultimate approaches for creating full and living engineered constructs in the biofabrication field. Interestingly, the process established to fabricate the cerebral blood vessel model using the CBVdECM and bioprinting induced the self-assembly of cells without any external stimuli. Thus, co-culturing HBMECs and HBVPs in CBV tissue constructs could create an anatomically relevant configuration of the lumen with an inner lining of HBMECs and an outer sheath of HBVPs, following multicellular self-assembly. Multicellular self-assembly was clearly achieved after culturing the printed CBV tissue construct for 7 d (Fig. 5A). We observed spontaneous compartmentalization with HBVPs wrapped around HBMECs (replicating the layered structure of the native BBB) following cellular interactions and migration. A fluorescence intensity plot demonstrated a clear positional distinction between the HBMECs and HBVPs (Fig. 5B to D). We attribute this self-organization of the cells to the synergistic effect of the biochemical niche and mechanical properties of the bioink and cellular forces exerted by the tubular structure [29–31].

Visualization of microfilaments in the vascular endothelium (F-actin) and endothelial cell-adhesion molecule (platelet endothelial cell-adhesion molecule-1 [PECAM-1]) revealed the vascular morphology of the mature CBV tissue construct (Fig. 5E). Additionally, VE-cadherin showed ample junctional marker expression, indicating vascular integrity in the cerebral blood vessel model (Fig. 5F). Finally, we measured the permeability of the CBV constructs by applying FITC-labeled dextran into the side chambers and perfusing them through the lumens of the constructs. To assess the effect of the self-assembled HBMEC and HBVP layers, we prepared a bare group (where no cells were encapsulated), a group with HBMECs, and an HBMEC + HBVP dual-layered group (Fig. 5H). The degree of diffusion to the abluminal side did not differ between the bare and HBMEC single-layered groups, whereas the HBMEC + HBVP dual-layered group showed substantially lower permeability than the single-layered group (Fig. 5I). The permeability of the CBV model can change as the cells undergo ECM remodeling during maturation. In the HBMEC-only group, the significant remodeling of the ECM by the cells increases the constructs’ permeability. In contrast, the self-assembled HBMECs + HBVPs group shows higher expression of junctional proteins such as VE-cadherin and PECAM-1, which create a tight barrier that counters the permeability increase caused by ECM remodeling. These data indicated that the self-assembled dual-layered cellular lining enhanced the barrier function of the CBV tissue constructs when compared with those of the single-layered and bare groups, making it a more reliable CBV model.

### The dual-layered CBV model demonstrates physiologically relevant neuroinflammatory responses

Finally, we observed neuroinflammation and subsequent responses in the CBV tissue constructs. A healthy BBB restricts molecular transport from the blood to neuronal sites in vivo and thereby protects the central nervous system from foreign or destructive molecules in the blood. Various cytokines can traverse the BBB and establish direct communication between the circulating immune system and the compartments of the central nervous system [2]. In the neuroinflammatory state, changes in the BBB enable intensive cellular trafficking, up-regulated cytokine release, and junctional breakdown. Neuroinflammation initiated by cytokines, including TNF-α and IL-1β, may be involved in BBB dysfunction and thereby promote the advancement of Alzheimer’s disease by fostering amyloid-β peptide accumulation [32]. Therefore, to evaluate the developed cerebral blood vessel model, we induced neuroinflammation with the matured constructs by treating them with representative neuroinflammatory cytokines such as TNF-α and IL-1β.

**Fig. 6.**
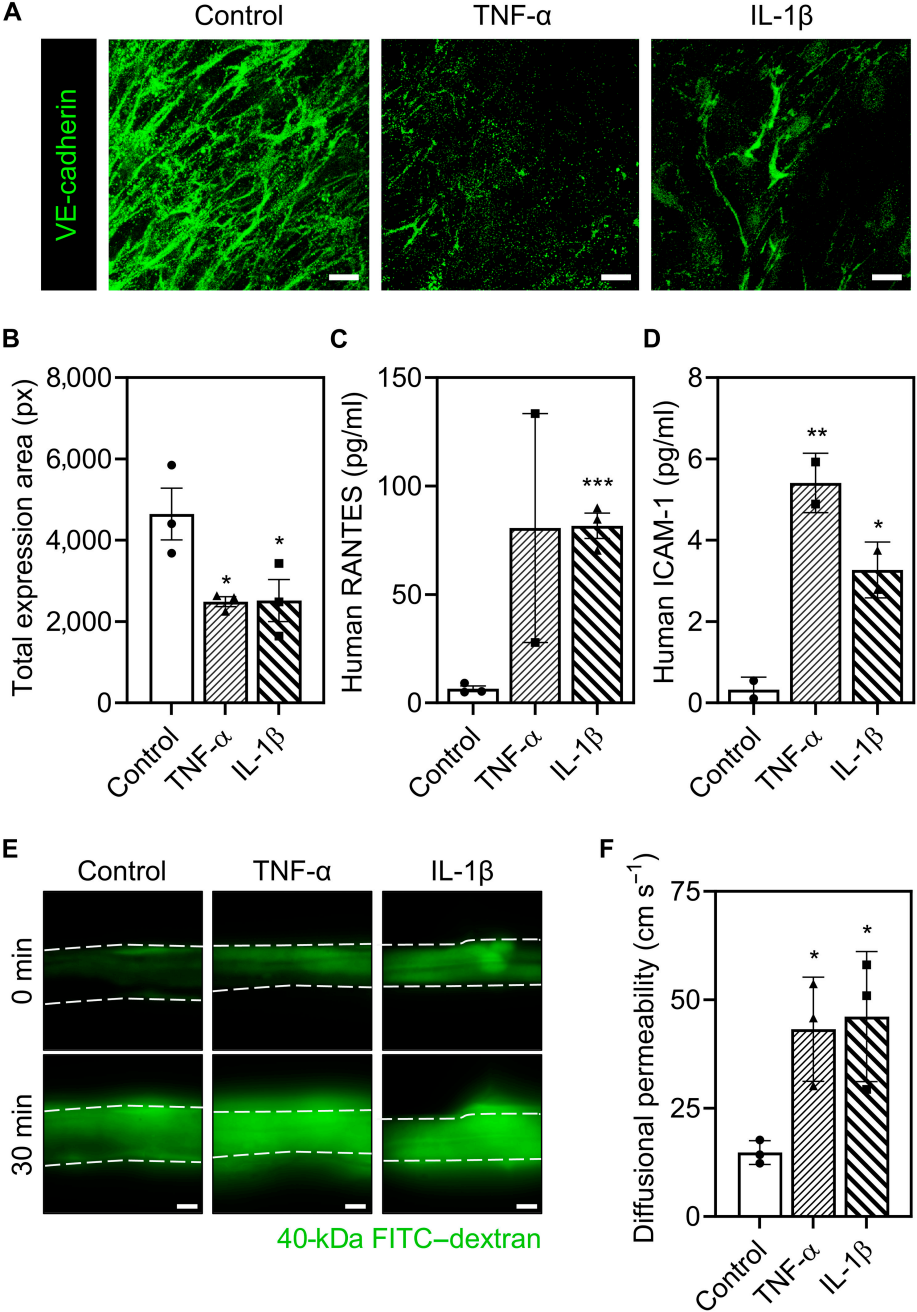
Neuroinflammatory responses observed with the cerebral blood vessel model. (A) Immunofluorescence staining showing junctional down-regulation during inflammation (scale bars: 20 μm). (B) Quantification of the total area of neuroinflammatory cytokine (RANTES) expression and adhesion molecule (intercellular adhesion molecule 1 [ICAM-1]) expression for the samples presented in (A). Data represent mean ± SEM (*n* = 3 per group; *P < 0.05; one-way ANOVA). (C and D) Up-regulation of RANTES (C) and ICAM-1 (D) after inducing inflammation in cerebral blood vessel constructs. Data represent mean ± SD (*P < 0.05; **P < 0.01; ***P < 0.001; one-way ANOVA). (E) Permeability assay: increased abluminal diffusion of FITC-labeled dextran during neuroinflammation of cerebral blood vessel constructs (green: 40-kDa FITC–dextran; scale bars: 200 μm). (F) Calculated diffusional permeability of the samples shown in (E), indicating that neuroinflammation increased permeability (10-kDa FITC-labeled dextran). Data represent mean ± SD (*n* = 3 per group; *P < 0.05; unpaired Student *t* test).

Significant junctional down-regulation, a representative form of the disruptive changes that occur during neuroinflammation, was validated by immunofluorescent staining for VE-cadherin and quantifying the total expression area (Fig. 6A and B). We observed expression changes in 2 representative neuroinflammatory cytokines: RANTES and ICAM-1 (Fig. 6C and D). RANTES is a proinflammatory chemokine that plays a role in immune modulation by attracting immune cells (including monocytes, granulocytes, and T cells) to sites of inflammation [33]. ICAM-1 mediates cellular inflammation and is potentially modulated by DJ-1 and α-synuclein (2 proteins closely linked to Parkinson’s disease), particularly during neurodegenerative processes that extend beyond the regulatory impact on endothelial functions [34]. Accordingly, RANTES and ICAM-1 are potential inflammatory biomarkers for detecting Parkinson’s disease. In our model, inducing neuroinflammation by treatment with TNF-α and IL-1β markedly increased RANTES and ICAM-1 secretion. In addition, we observed markedly increased diffusional permeability in the TNF-α- and IL-1β-treated groups, representing the final form of disruptive change that occurs during neuroinflammation (Fig. 6E and F). In conclusion, we validated the performance of our CBV tissue construct (which can be considered a promising tool for modeling neuroinflammation) after successfully demonstrating inflammatory cytokine up-regulations and junctional breakdown in vitro.

## Discussion

In this study, the bioprinted tubular cerebral blood vessel structure simultaneously induced the multicellular self-assembly of HBMECs and HBVPs into anatomically relevant layered arrangements, where the distinct endothelial membrane was tightly surrounded by pericytes. We also demonstrated the diffusional permeability of the self-assembled CBV constructs and their ability to accurately replicate neuroinflammation, as evidenced by increased cytokine expression and the breakdown of endothelial tight junctions.

The BBB is a dynamic interface that separates the neural environment from the bloodstream and responds to shear stress induced by blood flow. Recent in vitro human BBB models have acknowledged and replicated this aspect by employing fluid flow to emulate dynamic circulatory conditions [35]. However, owing to the inherent characteristics of microfluidic device manufacturing processes, the sandwich-like and planar parallel configurations of conventional BBB models often exhibit prismatic geometry, which disturbs the generation of uniform shear stress with such models. Consequently, enhancing the 3D arrangement in BBB models can establish a tubular design with cylindrical channels, which enables consistent shear stress along the entire inner surface [36].

Previous data demonstrated the effectiveness of 3D bioprinting technology with a multihead printing system in precisely and reproducibly fabricating tissue-specific shapes in a single step [37,38]. In particular, coaxial printing can be used to simultaneously coextrude multiple materials and generate 3D tubular structures, such as vasculature, while the tubular geometry serves as a physical cue to promote the self-organization of the cells [10,12,39,40]. Although the aorta and BBB share common structural and developmental traits as vascular entities, they serve distinct physiological functions and have unique characteristics tailored to their roles [41]. Unlike most blood vessels, which are largely impermeable, the BBB demonstrates selective permeability; thus, the BBB acts more as a discerning sieve than an absolute blockade [41]. This distinct functional difference reflects the intricate structure of the BBB, which consists of multiple compartmentalized layers composed mainly of endothelial cells, pericytes, astrocytes, and a basement membrane, wherein the endothelial membrane and basement membrane are only 0.3 to 0.5 μm and 20 to 200 nm thick, respectively [42,43]. Because previous top-down fabrication approaches have frequently shown difficulties in replicating these multicellular layers in the desired dimensions, bottom-up strategies that rely upon the self-assembly and self-organizing properties of cells have been regarded as an ideal approach [44–46].

We used a 3D coaxial printing technique to develop a new in vitro cerebral blood vessel construct composed of self-assembled, tubular freestanding microvessels containing HBMECs and HBVPs. The freestanding ability facilitates the investigation of molecular transport between the blood and neurons when studying the mechanism of neuroinflammation, unlike in previous models that require a supporting matrix [26,27,45]. Owing to the simultaneous multicellular self-assembly, we were able to effortlessly fabricate a multilayered, tubular in vitro BBB model that recapitulated the hierarchical native BBB structure in a more physiologically accurate manner, when compared with the self-assembled spheroid model, where the HBMECs and HBVPs were arranged in reversed directions [44,47], in a much simpler way than previous approaches, such as combining coaxial printing and electrospinning [48]. We believe that this morphogenic phenomenon can be ascribed to the CBVdECM, as biomaterials can guide morphogenesis in in vitro cell and tissue models [49].

This model could be used to develop a neurodegenerative disease model that is useful for mechanistic studies of neuroinflammation-associated pathology, including BBB disruption and α-synuclein accumulation in Parkinson’s disease. An attractive prospect for refining the present model is the inclusion of additional types of cells involved in neuroinflammation (such as astrocytes, neurons, monocytes, and leukocytes) to increase the reliability of mechanistic studies. In addition, developing a robust methodology for gauging the permeability of a 3D tubular construct (such as that presented here) is promising, which could be instrumental in assessing the vascular functionality of the evolving model. In the future, we are committed to conducting a more comprehensive exploration of how the CBVdECM drives optimal CBV function.

## Data Availability

All data are available in the main text or the supplementary materials.
