# Supplementary material for: Cerebrovascular-Specific Extracellular Matrix Bioink Promotes Blood–Brain Barrier Properties
Source: Biomater Res. 2024 Dec 5;28:0115. doi: 10.34133/bmr.0115 (PMC11617618; doi:10.34133/bmr.0115)

Supplementary Materials

**Cerebrovascular-specific extracellular matrix bioink promotes blood-brain barrier properties**

Hohyeon Han†, Sooyeon Lee†, Ge Gao, Hee-Gyeong Yi, Sun Ha Paek* and Jinah Jang*

Figs. S1

Tables S1

Movies S1

**
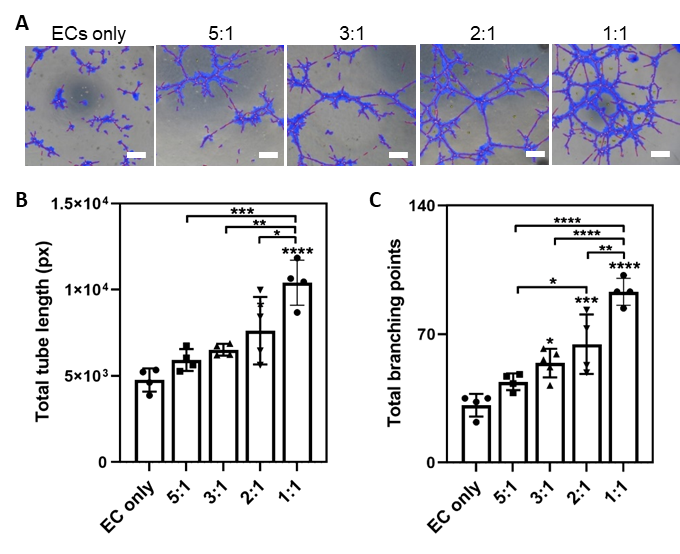
Fig. S1.** Co-culture ratio optimization of HBMECs: HBVPs by tube formation assay.
Effect of the co-culture ratio of HBMECs:HBVPs (2 × 10^5^ cells/ml) on *in vitro* vascularization was examined with tube formation assay. (A) Representative bright field image (blue dot: recognized area of the tube, scale bar: 500 µm). (B) Total tube length and (C) Total branching points per field were quantified by WimTube software (n=4).

**Table S1.** Mean and s.d. (n = 5) of remaining content of DNA [ng/mg] and GAGs [%] in two types of dECM biomaterials.

|  | Biomaterial | | | |
| --- | --- | --- | --- | --- |
| Component | BdECM | | VdECM | |
|  | mean | s.d. | mean | s.d. |
| DNA | 29.19 | 0.40 | 1.68 | 1.48 |
| GAGs | 84.65 | 2.51 | 72.99 | 4.33 |

**Movie S1.** One-step fabrication of supporting chamber and CBV construct


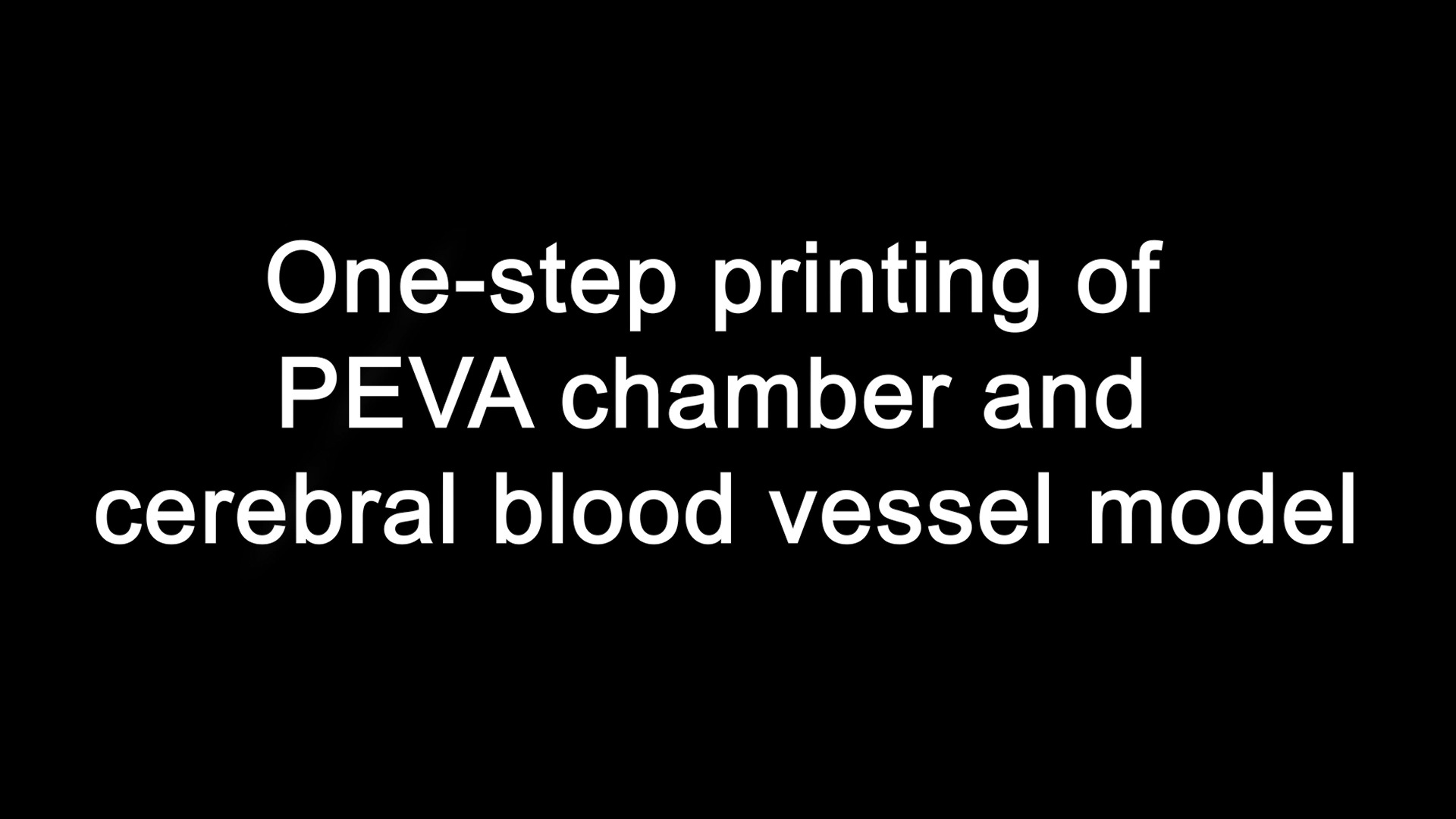

Supplement: Supplementary 1 — Fig. S1 Table S1 Movie S1 [file bmr.0115.f1.zip › Supplemental file (20241011).docx]
